# Before it disappeared: ethnobotanical study of fleagrass (*Adenosma buchneroides*), a traditional aromatic plant used by the Akha people

**DOI:** 10.1186/s13002-018-0277-9

**Published:** 2018-12-21

**Authors:** Yi Gou, Ruyan Fan, Shengji Pei, Yuhua Wang

**Affiliations:** 1 Department of Economic Plants and Biotechnology, Yunnan Key Laboratory for Wild Plant Resources, Kunming Institute of Botany, Chinese Academy of Sciences, 132# Lanhei Road, Kunming, 650201 China; 2 University of Chinese Academy of Sciences, Beijing, 100049 China

## Abstract

**Background:**

Fleagrass, *Adenosma buchneroides*, is an aromatic perennial herb that occupies an important position in the life of the Akha people. They regard it as a tribal symbol and a gift of love. Fleagrass also has many medicinal uses, and there is considerable potential for its development as an insect repellent. Traditionally, Akha people plant it in swidden fields, but there are few swidden fields in China now. Therefore, the first question this study aims to answer is as follows: how is fleagrass planted and utilized now? At present, fleagrass is only reported to be used by Akha people in Mengla. We also try to understand the following questions: Is fleagrass used in nearby area? If so, how is fleagrass used in nearby area? Furthermore, why is fleagrass used in that way?

**Methods:**

From August 2016 to July 2018, field surveys were conducted six times. The ethnobotanical and ethnopharmacological uses of *A*. *buchneroides* in 13 Akha villages were investigated by means of semi-structured interviews. We assessed the responses of a total of 64 interviewees (32 men and 32 women; mean age, 58.6) from the Xishuangbanna Dai Autonomous Prefecture, southwest China, and from Phongsaly Province, Laos. To explain the bases for the ethnobotanical uses of fleagrass, we used Google Scholar, Web of Science, and China National Knowledge Infrastructure to review the bioactivities of the chemical constituents of *A*. *buchneroides.*

**Results:**

With the vanishing of swidden agriculture and the development of modern products, fleagrass cultivation is disappearing in China. However, most Akha people in Xishuangbanna still remember and yearn for its traditional uses, and Akha people in a nearby area (northern Laos) continue to plant and utilize it. We documented ten uses of *A*. *buchneroides* within five discrete categories*.* The whole plant of fleagrass has a distinct strong aroma, of which Akha villagers are particularly fond. Akha villagers mostly use this aromatic property as a decoration, perfume, and insect repellent. *A*. *buchneroides* is also used as a condiment and for medicinal and ritual purposes, including its use as a cure for insect bites, headaches, influenza, and diarrhoea, and as a part of pray ritual for a bumper harvest. From our literature review, we identified many major chemical compounds contained in the essential oil of *A*. *buchneroides*, including thymol, carvacrol, 3-carene, and *p*-cymene, which have insecticidal or insect-repellent, antimicrobial, and anti-inflammatory properties.

**Conclusion:**

Fleagrass is an aromatic plant that is widely used by Aka people. Its chemical composition also has a variety of biological activities. With the vanishing of swidden agriculture and the development of modern products, fleagrass utilization in China is disappearing and its cultural importance is reduced. However, its economic and medicinal value is assignable.

**Electronic supplementary material:**

The online version of this article (10.1186/s13002-018-0277-9) contains supplementary material, which is available to authorized users.

## Introduction

Vector-borne diseases pose a significant threat to human life and welfare and account for approximately 17% of the global burden of infectious diseases, causing more than one million deaths each year [1]. More than 80% of the world’s population live in areas with at least one major vector-borne disease risk, with more than half of the population facing two or more such risks [1]. For most vector-borne diseases, prevention by targeting vectors is the first and best approach. Use of long clothing and topical repellents for personal protection is one of proven effective vector control approaches. When considering the adverse environmental effects and the development of resistance to synthetic repellents, there is currently a greater requirement for plant-based repellents [2].

People have been combatting insects for thousands, even millions, of years. Several species of primates have been observed rubbing plants to repel insects [3]. The earliest recorded human use of plants as a repellent can be traced back to BC. Herodotus recorded that Egyptian fishermen burn castor oil in oil lamps to repel mosquitoes [4]. In the Chinese ancient book ‘Zhou Li’ (one of four extant collections of ritual matters of the Zhou Dynasty, 1046 B.C. to 256 B.C.), there is a record of burning plants to fumigate insects [5]. In the Arribes del Duero (western Spain), local residents reported 27 traditional uses of 22 plants to prevent or control mosquitoes, flies, and other insects. Of the 27 reported remedies, inhabitants of the area continue to use 16 (59%), mainly against mosquitoes and houseflies [6]. Boer et al. conducted structured interviews in 66 villages of 17 ethnic groups, throughout the Lao People’s Democratic Republic. A total of 92 plant species was recorded to repel or kill hematophagous arthropods, including mosquitoes, bedbugs, human lice, mites and ticks, fly larvae, and blood-sucking leeches [7]. In Africa, some places are the hardest hit areas by insect-borne diseases, where local residents still apply plants in traditional ways as an effective and economical means of insect control [8, 9].

Peasants of all regions knew well how to use specific local plants to protect themselves against insects. They named some of them according to the insect for which they were used to repel. For instance, almost all species in the genus *Polygonum* have the same local name, kirburohi, or ‘flea herb’ in Estonian scientific nomenclature, which is because these species are used exclusively against human fleas. The whole plant was cut, dried, and then placed on the floor or in the bed to kill fleas [10]. In addition, some special plants are endowed with special cultural meanings. A typical example is *Artemisia vulgaris*, which is hung on the door as part of a ceremony to drive away pests and diseases for the Dragon Boat Festival. In rural China, the burning of bundles of dried *A. vulgaris* to produce smoke had been used to repel insects for centuries [11]. After the Dragon Boat Festival, the weather starts to heat up, and the snake worms and ants began to move. Therefore, people usually put a branch of *A. vulgaris* at their doorsteps to ward off diseases, prevent mosquitoes, and ward off evil spirits. Although modern products have replaced traditional protection, this ritual continues today.

In Mengla County of China, there is also a plant named ‘fleagrass’, *Adenosma buchneroides*. Similar to the *Polygonum* in Estonia, fleagrass is an insect-repellent plant that is used by the Akha people. However, fleagrass plays a broader and more important role in the culture of Akha. Akha people wear it on the head every day, because they like the smell and regard it as the symbol of Akha [12]. The smell is also good for relaxing and comforting the body. In traditional Akha medicine, the whole plant has medicinal value in anti-rheumatic, diminishing swelling, dissipate stasis, and analgesia [13]. The essential oil of fleagrass exhibited a good reduction of the population of adult insects [14]. Chemical constituents of the essential oil of fleagrass have been identified, with 39 chemical compounds reported. The main constituents include thymol, carvacrol, 3-carene, *p*-cymene, and β-phellandrene [12, 15]. These chemicals show antimicrobial, antioxidant, phytotoxic, and insecticidal activities [16–19].

**Fig. 1:**
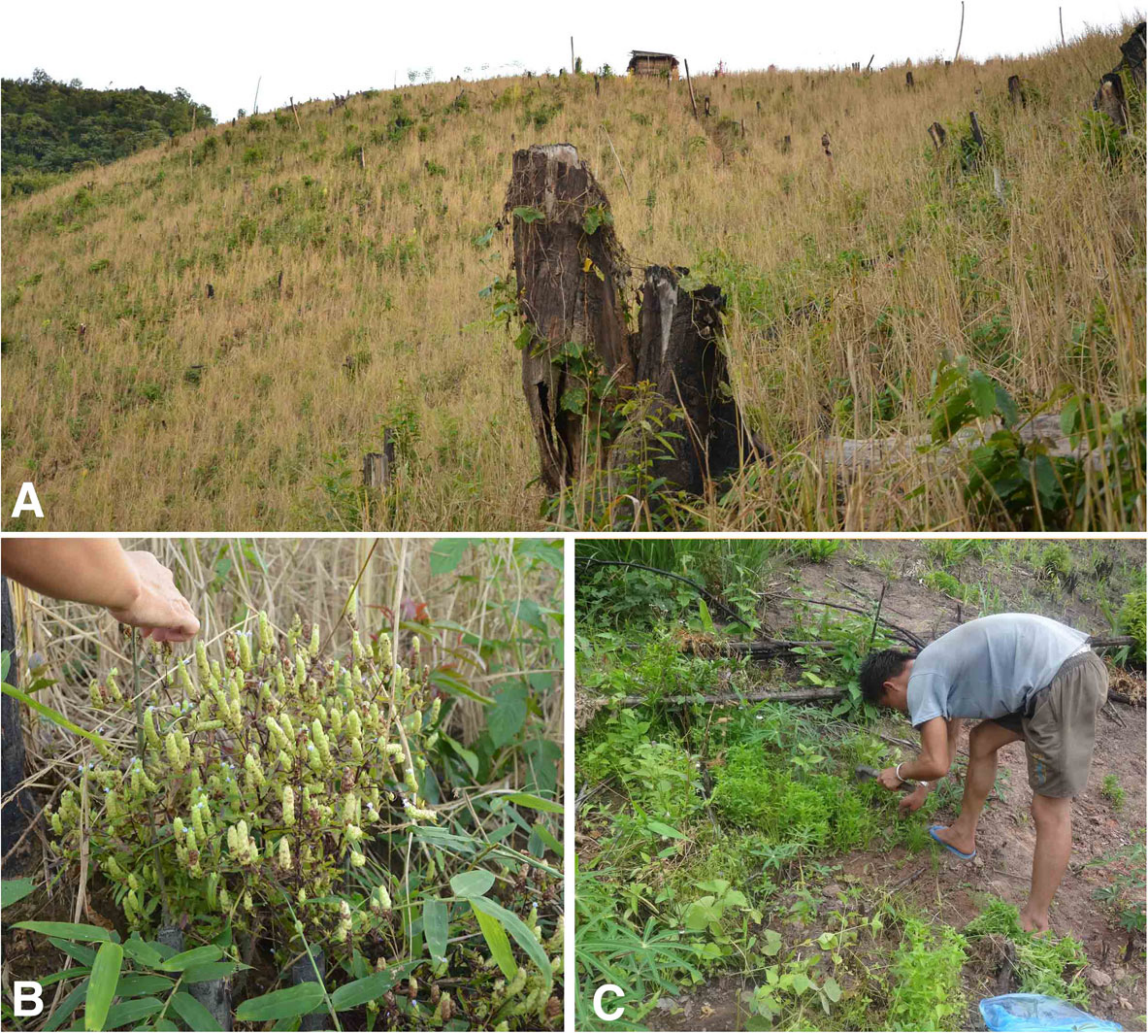
Fleagrass habitat and harvest. **a** The habitat of fleagrass. **b** The harvest of ripe fleagrass. **c** The harvest of green fleagrass (photographed by YG and YW, made by A. B. Cunningham)

Botanically, fleagrass is an aromatic perennial herb with an erect stem and many branches that can grow to a height of 40–45 cm. The stems are angulate, bearing paired leaves, each of which is 1.2 × 2 cm in size and ovate-oblong in shape. The purple flowers form terminal spikes that bloom from October to November. The fruit is an oblong capsule that dehisces into four straw-coloured lobes when mature. The fruiting spike can be as long as 5 cm. The numerous minute ovate seeds mature in December (Fig. 1).

**Fig. 2:**
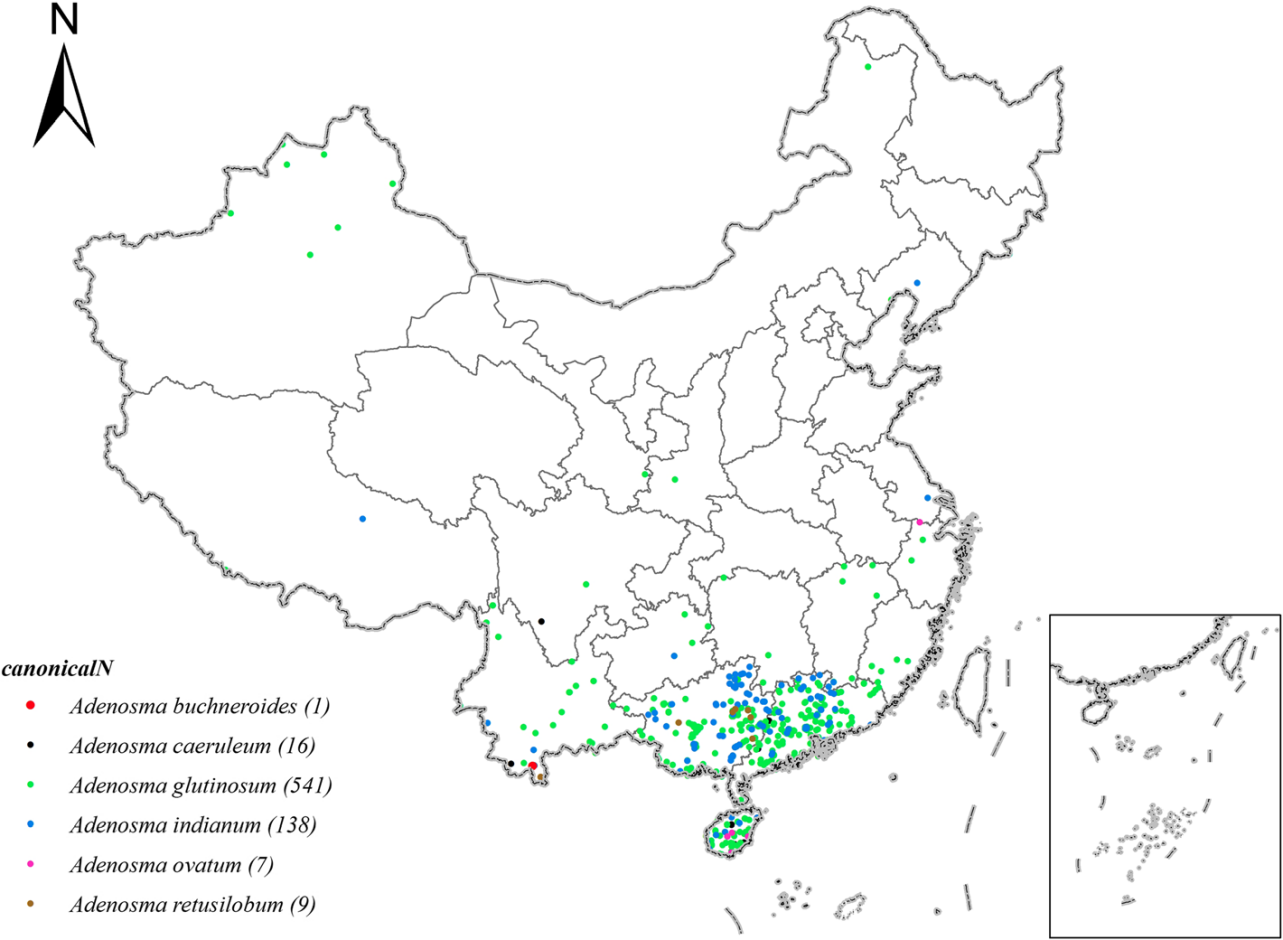
Distribution of *Adenosma* species in China (data from GBIF, CVH, NSII)

Interestingly, although fleagrass is naturally distributed in Vietnam [20], there is no wild fleagrass in China. There are five other *Adenosma* species that are naturally distributed in China (Fig. 2), and four species are recorded as Chinese herbal medicines (*A*. *caeruleum*, *A*. *glutinosum*, *A*. *retusilobum*, and *A*. *indianum*) [13, 21].

Akha people in Mengla planted fleagrass together with rice in swidden field [12], but there are few swidden fields in China now. Therefore, the first question this study aims to answer is as follows: How is fleagrass planted and utilized now? At present, fleagrass is only reported to be used by Akha people in Mengla. We also try to understand these questions: Is fleagrass used in nearby areas? How is it used in nearby area? Furthermore, why is it used in that way?

## Materials and methods

### Study area

**Fig. 3:**
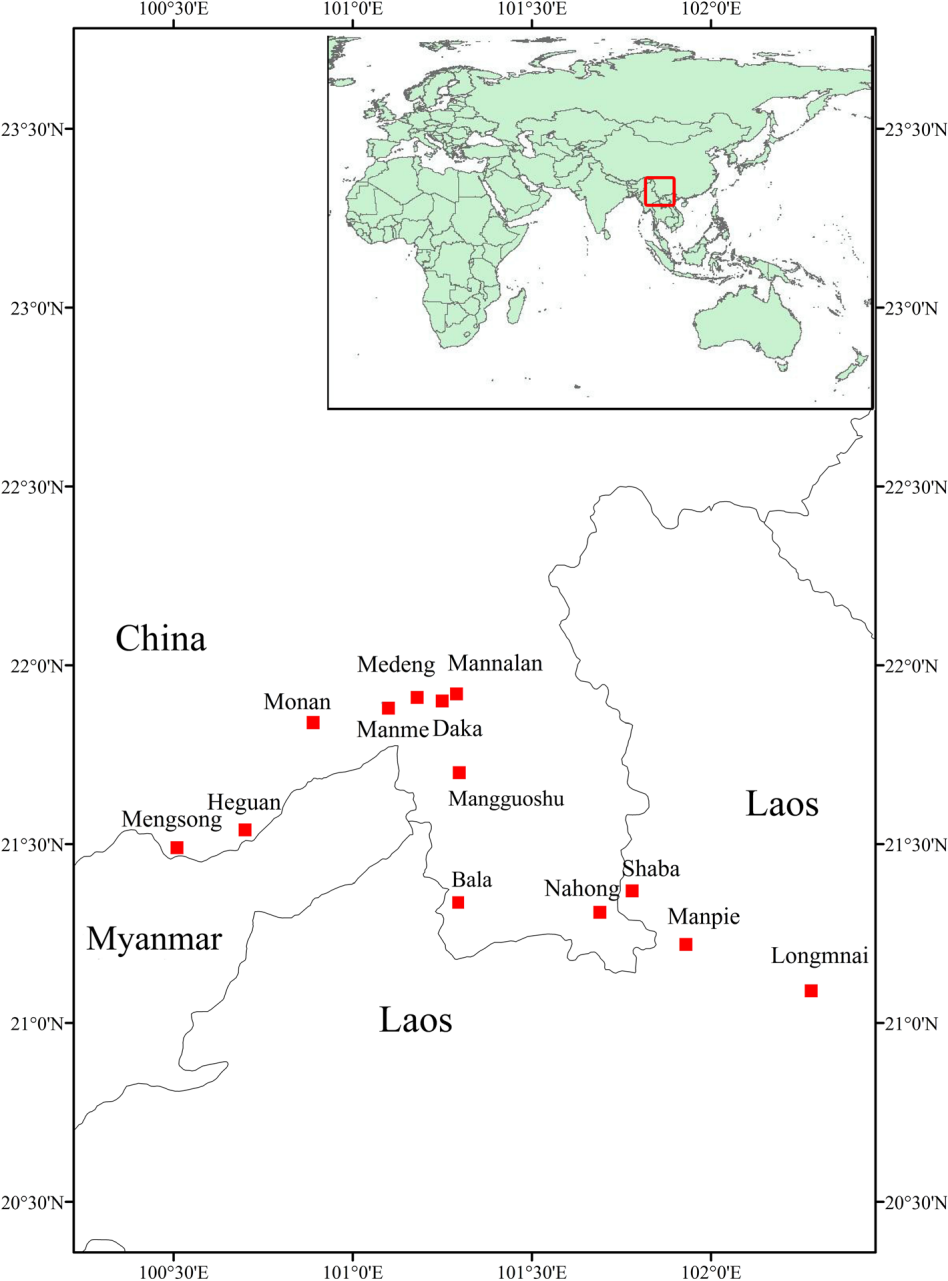
Study villages of China and Laos (made by Meng Wang)

The study was carried out in 13 villages of two neighbouring areas, Xishuangbanna Prefecture (China) and Phongsaly Province (Laos), which have similarities in climate, vegetation, and culture. These areas are situated in the tropical monsoon forest zone of southeastern Asia within latitudes 20–24° N and longitudes 98–104° E (Fig. 3).

Xishuangbanna Dai Autonomous Prefecture is located in the southern-most part of Yunnan Province, southwestern China, bordering Laos and Myanmar, covering a total area of 19,125 km^2^ and having a population of approximately 1.18 million [22]. The Mekong River runs through the region from northwest to southeast. The climate is subtropical with a rainy season between May and October and a dry season lasting from November to April. Annual precipitation varies from 1200 to 1900 mm in the valleys and uplands, of which 84% occurs during the rainy season and 16% in the dry season [23]. The Hani people are the second largest ethnic group in Xishuangbanna Prefecture, comprising approximately 19% of the population and the majority of Hani people here are Akha, who live in the mountain and mid-mountain areas [24].

Phongsaly Province is one of the remotest Lao PDR provinces. It is dominated by a rugged, mountainous terrain and supports an abundance of thick forests and fast-flowing rivers. Most of the land lies between elevations of 500 and 1500 m, which moderates the heat of the surrounding areas and makes the climate suitable for trekking and other physical activities. Agriculture is the mainstay of the people of the province. The March 2015 census put the population of the province at 177,989. There are 13 minority ethnic groups, including the Khammu, Thai Dam, Thai Daeng, Yao, Leu, Hor, Hmong, Akha, Yang, Bid, and Lolo. Phongsaly is located between Yunnan (China) and Điện Biên Province in Vietnam. There is considerable communication among the groups here, particularly within the same ethnic tribe [25].

### Ethnobotanical surveys

Field surveys were conducted in August, September, and November 2016, January and May 2017, and July 2018. Ethnobotanical data were collected through semi-structured interviews with informants in Xishuangbanna Dai Autonomous Prefecture and Phongsaly Province.

With the ongoing disappearance of swidden agriculture, the resource of fleagrass in China is decreasing, and knowledge about fleagrass is currently restricted to only few people. Among these, the majority are traditional healers and herbalists, and therefore, for our survey work, we selected eight villages that are inhabited by traditional healers and herbalists. In each village, we held the first meeting with the village chief and his advisors to inform them of the purpose of our work. The village head would consent to the research and recommend traditional healers or herbalists to be interviewed for the study. Furthermore, we also interviewed some other informants who were elderly villagers or had a sound knowledge of fleagrass. With the assistance of a guide who can speak Lao, Akha, and Chinese, we also selected three Akha villages in Phongsaly Province, Laos, where they still cultivate fleagrass. In the villages of Laos, we selected informants randomly. A voucher specimen (KUN: 1341141), which was deposited in the herbarium of the Kunming Institute of Botany, China (KUN), was used as a representative sample of the plant for identification by respondents. Informants were asked whether they were able to identify the fresh plant. If they recognized the fleagrass, they were asked to provide information about their knowledge of the plant, the local name, medicinal use, place and mode of collection, preservation, plant preparation, and administration.

In total, we interviewed 64 people from 13 localities: eight in Xishuangbanna and three in Phongsaly. We obtained responses from a total of 64 interviewees from Xishuangbanna Dai Autonomous Prefecture, southwest China, and Phongsaly Province, Laos. The 64 respondents fell into three age groups: (1) 20–39, (2) 40–59, and (3) ≥ 60 years old, with an average age of 58.6 years. Most respondents (53%) were aged above 60 years, 36% were between 40 and 59 years old, and 11% were between 20 and 39 years old. There were 32 (50%) female respondents and 32 (50%) males. The occupation of the respondents included doctor, herbalist, witch, and village head (Additional file 1).

### Data analysis

Frequency of citation (FC) is the number of informants who refer to each usage; frequency of current use (FCU) is the number of informants who continue to use them today.

### Literature review

To explain the ethnobotanical knowledge of fleagrass, we surveyed the literature relating to fleagrass chemical composition and selected the constituents that are related to the uses of fleagrass. For this purpose, literature searches were conducted using Google Scholar, Web of Science, and CNKI (China National Knowledge Infrastructure). We wanted to identify scientific publications and patents describing in vitro or in vivo studies of fleagrass and the bioactive components relevant to its uses and applications. The search for biological activity was conducted individually for the species and each chemical constituent. We combined the following keywords using the Boolean operators ‘AND’ and ‘OR’: ‘*Adenosma buchneroides*’; ‘chemical compound’ (e.g. thymol); ‘biological’; ‘pharmacological’; ‘activity’; ‘bioactivity’.

## Results and discussion

### Ethnobotanical usages of fleagrass

Fleagrass is known by Akha people with the vernacular name ‘Lao-wo-suo-du’, ‘Lang-suo-du’, and ‘La-sang-suo-du’. From respondents who have an ethnobotanical knowledge of fleagrass, we recorded ten uses within five categories (Table 1). These uses from the Akha people in Xishuangbanna and Phongsaly have similarities and differences.

**Table 1:** Different uses of fleagrass recorded among the Akha people in Xishuangbanna and Phongsaly

Parts used	Use category	Mode of preparation	Form of use	Purpose of use	Usage in China(C)/Laos(L)	FC	FCU
Aerial part	Personal	Placed in small bundles	Worn in ear-holes or on caps	Perfume; decoration	C,L	34	9
	Medicine	Placed in small bundles	Worn in ear-holes or on caps	Headache	C	11	
				Stuffy nose	C	2	
	Insecticide	Placed in small bundles	Hung on the window/placed on the floor or bed	Insect repellent	C	22	
	Ritual	Placed in small bundles	Hung on the wall of granaries	When praying for a bumper harvest	L	17	17
Whole plant	Medicine	Boiled in water as an infusion	Drunk as an extract	Influenza	C	4	
				Diarrhoea	C	3	
	Insecticide	Boiled in water as an infusion	Body wash	Insect bites	C	7	3
				Insect repellent	C	5	3
		Planted around the house		Insect repellent	C	9	
Leaves	Medicine	Crushed leaves	Rubbed on a wound	Insect bites	C	17	5
				Acne	C	1	
	Condiment		As a fragrance	Taste	L	12	12

**Fig. 4:**
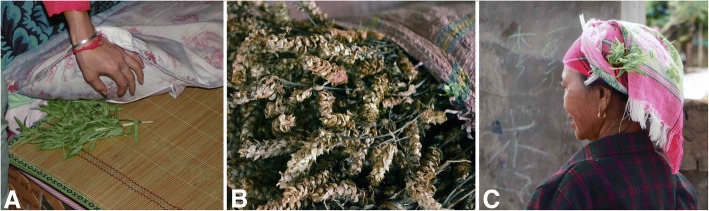
Fleagrass uses and dried plant. **a** Put fleagrass on bed. **b** Dried fleagrass. **c** Wear fleagrass on the head (photographed by YG and Changan Guo, made by A. B. Cunningham)

The whole plant has a fresh fragrance that is emitted when the living leaves are rubbed, and dried specimens retain their strong fragrance. Akha villagers are particularly fond of its scent. Both China and Laos Akha villagers put fleagrass in ear-holes or on caps as decorations or perfume (Fig. 4c). Some respondents also claimed that placing fleagrass in their ear-holes or on caps can relieve headaches. The scent is also helpful in relieving nasal congestion.

Akha villagers live in tropical and subtropical rainforests, where many types of insects are both a source of annoyance and can pose a risk to human health. As a preventative measure, in China, the Akha villagers place bundles of fleagrass in their rooms or spread fleagrass on beds to keep fleas or other insects away from human bodies (Fig. 4a). They also plant fleagrass around sheds in fields to repel insects. Fleagrass is also an important medicinal plant that is utilized by Akha people of China. They use fleagrass by making a potion for drinking to gain relief from headaches, influenza, or diarrhoea, which serves as a means of family health care in remote areas. They also crush fresh fleagrass and apply it to the skin to treat insect bites, as the sap aids in relieving itching and swelling. Furthermore, they make an infusion of fleagrass by boiling the grass in water and use this infusion to scrub and clean insect bite wounds. This infusion is also used in treating acne. In addition, they believe that when the body has been washed with this boiled preparation, they will not be bitten by insects.

In Laos, the Akha have unique uses of fleagrass, including consumption of the fresh leaves as a flavouring, because they like its aroma and piquancy. Fleagrass is also regarded as a ritual plant, and the Akha hang bundles of fleagrass in their granaries as an offering in hope of a bumper harvest.

Akha swidden farmers of China and Laos have similar cultivation experiences. They cultivate fleagrass together with upland rice as an inter-cropping system. Fleagrass seeds are planted simultaneously with dry rice seeds in April (the beginning of the rainy season), although in different holes in the fields. Fleagrass plant growth is slower than that of rice plants, and thus, fleagrass plants are always below a canopy of rice foliage, which inhibits fleagrass growth. Swidden farmers harvest rice from fields in October, at which time the fleagrass is flowering. With the rice harvested, the fleagrass is exposed to considerably more sunlight, and the open field conditions are favourable for pollination. When the fleagrass fruits are sufficiently mature, they are harvested for family use. However, with the continuing disappearance of swidden agriculture in China, there are only a few people who still plant fleagrass. Considering the requirement for shade, fleagrass is now planted together with tea trees. The Akha typically collect dried plants bearing seeds, the bundles of which are hung around their homes. The seeds are separated in the following year and subsequently planted. Generally, each family grows fleagrass annually in a small plot of upland rice.

### Cultural meanings and beliefs associated with fleagrass

The Akha people like the scent of fleagrass and often wear fresh or dried branches on their heads as decoration and perfume. Other ethnic groups, including other swidden groups such as the Jino, Yao, Miao, and Lahu, within the same area do not use fleagrass. The Akha people regard it as a tribal symbol, which indicates that they are different from other tribes. Because this kind of grass is only planted and used by the Akha people, when they wear the fleas on the head, they also have a sense of pride and belonging. Akha villagers make use of fleagrass as decorations and perfume in different styles according to age, sexuality, and tribal identity. Elders among the Akha villagers use fleagrass in small bundles to wear in their ear-holes or only in the right ear-hole as a decoration and perfume. Akha women wear a small bundle of fleagrass attached to their colourful woman’s cap. However, Akha men and little boys and little girls do not usually make use of fleagrass as a decoration.

Akha young men and women enjoy full social freedom. Each village has a ‘public house’ that is dedicated to providing space for young people to have parties and dates. Unmarried men and women can go to the public house to sing, dance, play, and talk to each other about love. When the young girl or boy comes to date, he or she will wear the fleagrass on his or her head as a sign. The girls put a bunch of fleagrass on the laces of their hats. The boys also wear a bunch of fleagrass on their ears. The scent of fleagrass makes the public house full of pleasant, fragrant tastes and provides a romantic atmosphere. When a young person falls for someone, they will also give ‘fleas grass’ as a gift of love.

Traditionally, the Akha have been a migratory people who practice swidden (slash and burn) agriculture. The mountain crops mainly include upland rice, corn, and cotton. The Akha plant fleagrass together with upland rice as an inter-cropping system. When the rice matures, the Akha harvest it and store the rice in the barn. At the same time, they pick up a bunch of fleagrass that is hanging outside the barn. This is a ritual that is used to pray for stored crops without insects and for the crops of the upcoming year to be bumper harvested without pests and disease.

### The rise and fall of fleagrass in China

Previously, there was no record of *A. buchneroides* in China, until Shen et al. (1991) found that fleagrass was introduced into swidden cultivating systems by Akha people in the Xishuangbanna area and served as a useful plant a long time ago [12]. Because the Akha people do not have tribal scripts, their knowledge of plant uses is passed on verbally, and thus, there is no written record about fleagrass in China in ancient times. When and how the Hani began their domestication of the plant in the area remains uncertain.

In China, someone said the Akha name of fleagrass ‘Lao-wo-suo-du’ means ‘grass that is from Laos’; ‘Lao wo’ means Laos in Chinese. Two informants reported a legend about the name. A short man brought fleagrass from Laos, and he sent it to the local people, but the local people did not take it because they did not know what it was. Then, the short man spread it everywhere, and there was abundant fleagrass in the field. Thus, they call it ‘Lao-wo-suo-do’. This legend may explain how fleagrass came to China from Laos. However, current wild distribution of fleagrass occurs only in Vietnam. It is still unclear how fleagrass spread from Vietnam.

*A*. *buchneroides* was first described in ‘Bulletin de la Société Botanique de Genève’ in 1913 by Bonati, who collected specimens in Vietnam and introduced *A. buchneroides* as an aromatic plant [15]. However, ‘Flore Du Cambodge, Du Laos, Et Du Vietnam’, ‘Checklist of Plant Species of Vietnam’, ‘Flora Yunnan’ and ‘Flora of Thailand’ do not recognize *A. buchneroides* as an individual species, they all regard it as a synonym of *A. indianum* [26–29]. *A. buchneroides* and *A. indianum* have the same Vietnamese name ‘Bobo’ [27]. *A. indianum* is also a Chinese medicine, and Chinese residents use it to cure colds, headaches, fevers, bellyaches, diarrhoea, cacochylia, and dermatitis [13]. These uses are similar with the uses for *A. buchneroides*. *A. buchneroides* and *A. indianum* have many similarities, but they also have some differences. The flowers of *A. buchneroides* and *A. indianum* densely connate and formatted into terminal or capitate spikes, but the inflorescence of *A. buchneroides* is longer than that of *A. indianum*.

Therefore, there are two hypotheses about the origin of fleagrass. Maybe it was disseminated from Vietnam through Laos to China. It is also possible that fleagrass was domesticated from *A. indianum*, and the inflorescence became longer in the process of cultivating. Testing these hypotheses requires more in-depth investigations and molecular evidence.

No matter how it begins, the current situation of fleagrass is not optimistic. With the continuing population growth and the introduction of cash crops and new policies, swidden agriculture in China is being replaced and is disappearing. Therefore, the resource of fleagrass is in danger of vanishing, particularly in China. Moreover, with improvements in the living standards of the Akha people, modern goods are increasingly replacing traditional materials. Therefore, the importance of fleagrass in Akha villages is declining, and with this decline, the traditional knowledge of fleagrass is becoming lost. Through field surveys, we accessed a total of 45 interviewees from Xishuangbanna Dai Autonomous Prefecture, Southwest China. There are 31 interviewees who have knowledge of a plant called fleagrass (or ‘lao-wo-suo-du’ in the Akha dialect) with morphological features as presented to them during the survey. Most of them have not cultivated fleagrass since 1990, when they began the large-scale planting of rubber. The significance of fleagrass in Akha traditional culture is therefore likely to die with reduced resources. An overwhelming majority of the young generation of Akha claim that they never see this plant and do not know about it. During the survey, we found only three people who continue to use fleagrass today. They save seeds and plant them in small scale, and they use fleagrass mostly in perfumes, to repel insects and alleviate insect bites, while other medical uses have been replaced by modern medicine.

Although fleagrass has died down from the daily life of the Akha people in China, they still remember the uses of fleagrass, and they yearn for it. When we showed the plant to the Akha people, they were happy and wanted to replant it. In the process of modernization, they accidentally lost fleagrass. However, fleagrass is able to occupy an important position again, as long as they are given the opportunity to replant it.

Compared to China, Akha of Laos still retain swidden agriculture, and the fleagrass resources are preserved. Considering the economic conditions and the convenience of life in Laos, the life of the Akhas is still heavily dependent on the resources of the forest plants, so the traditional knowledge of the fleagrass has been preserved. In Laos, each of the 19 interviewees we accessed know and use fleagrass now, and the mean age is 43 years old, which is younger than that of the respondents of China.

In conclusion, we think the vanishing of swidden agriculture and the development of modern products are the main reasons why fleagrass in China is disappearing. Nevertheless, we found that few Akha people of China continue to use fleagrass in perfume, to repel insects and to alleviate insect bites. Therefore, we think that fleagrass is effective at repelling insects and alleviating insect bites, but it is not widely known.

### The scientific basis of folk usages

Shen et al. [12] and Xu et al. [15] have examined the chemical constituents of the essential oil of fleagrass, identifying 26 and 34 chemical compounds, respectively. Most of the chemical constituents identified were common to both studies, and in total, 39 different constituents were recorded. However, the contents of the main compounds showed considerable variation, which could be attributable to differences in the materials examined. Shen et al. (1991) collected mature whole plants and dried plants that are harvested from swidden fields by Akha villagers [12], whereas Xu et al. collected the aerial parts of fleagrass planted in gardens [15].

Fleagrass is an important and useful aromatic plant with a distinct aroma. The main chemical constituents of the essential oil are terpenoids, which have distinct odours and bioactivities. We selected 23 compounds that have biological activities that are related to the ethnobotanical uses of fleagrass, as shown in Table 2. Thymol, carvacrol, 3-carene, *p*-cymene, β-phellandrene, α-phellandrene, cuminic acid, β-bisabolene, α-pinene, and limonene are the main constituents of the essential oil of fleagrass. The bioactivities of these constituents can be divided into six categories: antimicrobial, sterilization, insecticidal, insect repellent, anti-inflammatory, and anti-viral.

**Table 2:** Twenty-three compounds and their bioactivities related to ethnobotanical uses of fleagrass

Compound	Content % (Shen et al. 1991) [12]	Content % (Xu et al. 2005) [15]	Bioactivity	Reference
3-Hexenol	0.01	0.04	Insect pest control	[30, 31]
α-Pinene	0.36	1.24	Antibacterial; antifungal; insect repellent	[32–34]
Sabinene	0.01	–	Antibacterial; insect repellent	[35, 36]
β-Pinene	0.01	0.05	Antibacterial; antifungal; antidepressant; insecticide	[32, 33, 37, 38]
α-Phellandrene	11.86	0.11	Antimicrobial; insecticide	[39, 40]
3-Carene	0.36	1.67	Oviposition deterrent insect repellent; antibacterial	[17, 19]
*p*-Cymene	4.40	6.60	Oviposition deterrent; antibacterial; insecticide	[16, 41, 42]
Limonene	0.39	2.49	Oviposition deterrent; insecticide; insect repellent; antimicrobial	[42–44]
β-Phellandrene	11.86	–	Insecticide	[45]
Terpinolene	0.01	0.33	Insecticide; insect repellent	[46]
Linalool	0.04	0.05	Antimicrobial; insecticide; antidepressant; anticonflict effects	[38, 47–49]
4-Terpineol	0.06	0.08	Antimicrobial; insecticide; insect repellent; anti-inflammatory	[50–53]
α-Terpineol	0.08	0.13	Antimicrobial; anti-inflammatory	[47, 52, 54]
Cuminic acid	11.17	–	Fungicide	[55]
Carvone	0.03	0.02	Antimicrobial; insect repellent	[34, 43]
Carvacrol	0.78	34.78	Antimicrobial; anti-inflammatory; analgesic; insecticide	[16, 56, 57]
Thymol	52.48	0.70	Antibacterial; insecticide; antioxidant; antimicrobial; anti-viral	[18, 58–61]
β-Caryophyllene	0.08	0.17	Insecticide; anti-inflammatory; antibacterial	[37, 62]
β-Bisabolene	10.57	1.34	Synergistic bactericidal; insect repellent	[17, 63]
Farnesene	0.69	–	Insect repellent	[64]
Guaiol	0.57	–	Insecticide	[65]
Eugenol	–	0.02	Insecticide, insect repellent; antifungal; anti-inflammatory	[36, 66, 67]
Caryophyllene oxide	–	0.01	Anti-platelet aggregation; antifungal; insecticide	[46, 68, 69]

As shown in Table 2, the majority of these 23 compounds, including thymol, carvacrol, 3-carene, and *p*-cymene, have been proven to have insecticide or insect-repellent activities, by virtue of their odour or contact toxicity. Traditionally, Akha villagers placed bundles of fleagrass in their rooms or caps and utilized the aroma to repel insects. The Akha villagers of Laos hung fleagrass on the wall of granaries and planted it in the field to protect the grains from grain pests which benefits the crop harvest. Therefore, they used fleagrass to pray for a bumper harvest. However, the Akha live in a humid tropical mountainous region where insect bites cannot be completely avoided. Such bites are often accompanied by swelling, itching, and even secondary infection. Like many other traditional plants used to treat itching skin, the mechanism of fleagrass is used to treat insect bites. Nevertheless, the analgesic activity of carvacrol and the anti-inflammatory activity of several chemicals are helpful in relieving pain and preventing infection.

Some aromatic compounds, such as pinene, can be used as relaxants to reduce fatigue and thus may have a beneficial aromatherapeutic effect. Akha villagers are accordingly fond of using fleagrass as a fragrance. Thymol, one of the main constituents, exhibited high anti-viral activity, which may be useful in relieving headache, stuffy nose, and influenza.

Fleagrass is also used as a food condiment by the Akha of northern Laos. In addition to its odour and taste, we believe that this use may be related to the plant’s content of antimicrobial compounds. Temperatures in the regions inhabited by the Akha are generally high, and people like to eat raw materials; thus, there may be plenty of microorganisms in the food. The antibacterial and fungicide activities of fleagrass are helpful in keeping food fresh and safe to consume.

## Conclusion

Fleagrass is a versatile aromatic plant that is traditionally planted in swidden fields by the Akha people. With the vanishing of swidden agriculture and the development of modern products, fleagrass in China is disappearing, and its cultural importance has been reduced. However, its economic and medicinal value is assignable. On the basis of an ethnobotanical survey of fleagrass in northern Laos and in China, we documented the Akha villagers’ use of fleagrass as a perfume, insect repellent, condiment, herbal medicine, and ritualistic plant. To the best of our knowledge, we report here for the first time that Akha villagers crush fresh fleagrass and apply it to the skin to treat insect bites, and the Akha in northern Laos eat the fresh leaves of fleagrass as a flavouring. Furthermore, they hang bundles of fleagrass in their granaries as a ritualistic offering in the hope of a bumper harvest. From our literature review, we identified chemical constituents of the essential oil of fleagrass that have good bioactivities related to antimicrobial, sterilization, insecticidal, insect repellent, anti-inflammatory, and anti-viral properties. Overall, we consider that fleagrass is a multi-functional plant with tribal characteristics, particularly with respect to its use as an insect repellent and in the treatment of insect bites.

Although some of the aforementioned folk uses can be assigned tentatively to the bioactivities of certain chemical constituents that are found in fleagrass, further targeted research is warranted in this respect. As a traditional insect repellent, further in-depth research on fleagrass may help us to develop new, effective plant-based repellents. In addition, with continuing economic development and environmental changes, the resources and traditional knowledge of fleagrass are in danger of vanishing. As an important plant resource, we should focus on the protection and exploitation of fleagrass. We therefore highlight the need for further research that focuses particularly on the cultivation of fleagrass, the biological activities of its essential oil or extracts, the mechanisms underlying its various activities, and product development.

## Additional file

Additional file 1: Interview information about Fleagrass. (XLS 69 kb)

## Abbreviations

FC: Frequency of citation
FCU: Frequency of current use
